# Parkinsonism in Inherited Metabolic Disorders: Key Considerations and Major Features

**DOI:** 10.3389/fneur.2018.00857

**Published:** 2018-10-12

**Authors:** Nattakarn Limphaibool, Piotr Iwanowski, Marte Johanne Veilemand Holstad, Katarzyna Perkowska

**Affiliations:** Department of Neurology, Poznan University of Medical Sciences, Poznan, Poland

**Keywords:** inherited metabolic disorders, lysosomal storage diseases, mitochondrial dysfunction, neurodegenerative disorder, Parkinson's disease

## Abstract

Parkinson's Disease (PD) is a common neurodegenerative disorder manifesting as reduced facilitation of voluntary movements. Extensive research over recent decades has expanded our insights into the pathogenesis of the disease, where PD is indicated to result from multifactorial etiological factors involving environmental contributions in genetically predisposed individuals. There has been considerable interest in the association between neurological manifestations in PD and in inherited metabolic disorders (IMDs), which are genetic disorders characterized by a deficient activity in the pathways of intermediary metabolism leading to multiple-system manifestations. In addition to the parallel in various clinical features, there is increasing evidence for the notion that genetic mutations underlying IMDs may increase the risk of PD development. This review highlights the recent advances in parkinsonism in patients with IMDs, with the primary objective to improve the understanding of the overlapping pathogenic pathways and clinical presentations in both disorders. We discuss the genetic convergence and disruptions in biochemical mechanisms which may point to clues surrounding pathogenesis-targeted treatment and other promising therapeutic strategies in the future.

## Introduction

Parkinson's disease (PD) is one of the most common neurodegenerative disorders, with a substantial, worldwide growth in prevalence in recent years. It is a debilitating disorder, manifesting as reduced facilitation of voluntary movements attributed to a progressive loss of dopaminergic neurons in the substantia nigra pars compacta (SNpc) and the formation of intracellular inclusions containing aggregates of alpha-synuclein (a-syn) (1). Over the past decades, extensive research has expanded our insights into the pathogenesis of the disease. It has been suggested that PD's complex and multifactorial etiology results from environmental contributions in genetically predisposed individuals (2).

Inherited metabolic disorders (IMDs) are a complex and diverse group of genetic disorders characterized by the disruption of cellular biochemical functions and deficient activity in the pathways of intermediary metabolism. Consequential toxic substance accumulation or end-product deficiency leads to a multi-systemic clinical picture from liver dysfunction and cardiomyopathy to progression to encephalopathy, coma, or death if left untreated (3). The prevalence of newborn screening and implementation of advance treatment strategies have resulted in an expanding adult patient population, presenting with late neurological symptoms and movement disorders (4, 5). There has been considerable interest in the association between neurological manifestations and IMDs, as this can provide evidence regarding the underlying pathophysiology, potentially leading to advances in diagnosis and therapeutic strategies. This review highlights the recent developments in the convergence of parkinsonism in patients with IMDs, with the principal objective of improving the understanding of the overlapping pathogenesis and clinical features in both disorders (Tables 1, 2).

**Table 1:** Parkinsonian features in IMDs.

**IMD**	**Parkinsonian features**	**References**
Gaucher Disease	Earlier age of diagnosis Asymmetric resting tremor is the most common feature Severe motor impairment Impairment in memory, executive function, visuospatial abilities, accelerated cognitive decline Greater risk of dementia, cognitive decline, global cognitive impairment Non-motor symptoms: anosmia, dysautonomia, REM sleep disorder, depression, anxiety, psychotic features Increase in mortality risk	(6–15)
Niemann-Pick Disease	Motor symptoms: Dysarthria, tremor, rigidity, bradykinesia, postural instability, stooped posture, festinating gait, impaired fine motor skills, hypomimia Autonomic dysfunction: orthostatic hypotension, constipation, urinary urgency and impotence Sensory symptoms: hyposmia, Difficulty with concentration	(16, 17)
GM1 Gangliosidosis	Akinetic-rigid parkinsonism Features of immobile face, bradykinesia, short-stepped gait	(18–20)
Neuronal Ceroid-Lipofuscinoses	Bilateral rigidity Rigidity, bradykinesia, tremor	(21, 22)
**Disorders of metal metabolism**		
Wilson's Disease	Rigidity, bradykinesia, shuffling gait, hypophonic speech, hypomimia, micrographia Tremor may be resting, intentional, postural, or action-induced Most common form of tremor is an irregular, jerky, dystonic tremor	(23–26)
Hemochromatosis	Resting tremor, bradykinesia, rigidity Asymmetric onset Mixed resting and action tremor	(27–29)
**Disorders of amino acid metabolism**		
Phenylketonuria	Rigidity and bradykinesia Tremor presenting both at rest and on action	(30, 31)
Maple Syrup Urine Disease	Bradykinesia on finger tapping No resting tremor	(32)
Methylmalonic acidemia	• Positive correlation between serum MMA and severity of neuropathic pain	(33)
**Inherited mitochondrial disorders**		
Masked faces, rigidospasticity, slowed movements, gait disturbances Mental impairment		(34)

*IMD, inherited metabolic disorder; REM, rapid eye movement; MMA, Methylmalonic acid*.

**Table 2:** Pathomechanism of neuronal damage contributing to parkinsonism in IMDs.

**IMD**	**Pathomechanism of neuronal damage**	**References**
Gaucher Disease	GBA1 mutations 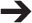 reduced GCase levels:	(35–44)
	Accumulation of GlcCer 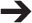 destabilization of a-syn tetramers, increase susceptibility of dopaminergic neurons to cytotoxic exposure to pathologic a-syn fibrils 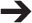 neuronal cell death Enhancement of the propagation of a-syn aggregations among neural cells 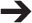 progression of PD	
	Parkin occupied by the degradation of misfolded GCase 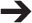 accumulation of other natural Parkin substrates 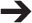 damage dopaminergic neuronal	
Niemann-Pick Disease	Dysregulated cholesterol trafficking 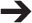 Cholesterol deposition in neurons	(45)
**Disorders of metal metabolism**		
Wilson's Disease	Excess Cu causes degeneration of dopaminergic neurons via several suggested pathways:	
	Interaction between Cu and a-syn 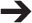 increased oxidative stress, production of toxic oligomers which result in membrane breakage, enhancement of a-syn fibrillation Ceruloplasmin deficiency 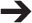 Excess free Cu 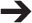 a-syn bound to free Cu increases availability of iron 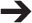 oxidative damage to SNpc and dopaminergic neurons by free radicals generated from both Cu and iron	(46–52)
Hemochromatosis	Excess iron causes degeneration of dopaminergic neurons via several suggested pathways:	(53–56)
	Enhancement of oxidative stress Generation of ROS and enhancement of protein aggregations 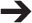 proteasome dysfunction 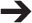 impairment of ubiquintin-proteasome system Promotion of a-syn aggregation and cell-to-cell transmission Induction of alterations in Parkin solubility 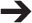 Parkin aggregation, depletion of functional Parkin 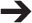 reduced proteasomal activities	
**Disorders of amino acid metabolism**		
Phenylketonuria	Reduction in brain tyrosine 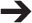 depletion of dopamine production Phe exposure in early life may cause adverse effects to brain and dopaminergic development Chronic Phe exposure associated with oxidative stress 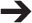 neuronal oxidative stress and tissue damage	(31, 57–60)
Maple Syrup Urine Disease	Early onset of encephalopathy 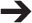 permanent brain damage Neuronal loss in pontine nuclei and SNpc	(32, 61–63)
	Accumulation of MSUD metabolites 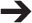 block respiratory chain 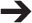 inhibition of brain energy metabolism Reduction in dopamine levels	
Methylmalonic acidemia	Methylmalonic acid accumulation 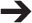 mitochondrial oxidative damage, oxidative stress enhancement 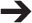 Neuronal apoptosis	(64, 65)
**Inherited mitochondrial disorders**		
Complex I deficiency in PD brain within the substantia nigra 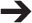 Oxidative damage from enhanced ROS production 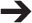 damage dopaminergic neurons Genetic overlap between PD and mitochondrial dysregulation: Under-expression of genes regulating pyruvate metabolism and ETC in the dopaminergic neurons of SNpc of PD patients Mutations in PINK1 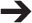 reduce energy production 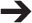 death of dopaminergic neurons Impaired degradation of MIRO 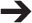 defective clearance of damaged mitochondria		(66–76)

*IMD, inherited metabolic disorder; GCase, glucocerebrosidase; GlcCer, glucosylceramide; a-syn, alpha-synuclein; PD, Parkinson's disease; Cu, copper; SNpc, substantia nigra pars compacta; ROS, reactive oxygen species; Phe, phenylalanine; MSUD, maple syrup urine disease; ETC, electron transport chain*.

## Inherited metabolic disorders

### Lysosomal storage diseases

#### Gaucher disease

Gaucher Disease (GD), a prevalent inherited lysosomal storage disease (LSD), is caused by deficient glucocerebrosidase (GCase) activity due to mutations in the coding gene (GBA). The resulting accumulation of glucosylceramide (GlcCer) in lysosomes of reticuloendothelial cells leads to complex systemic manifestations involving the liver, spleen, bone marrow, and to progressive neurodegenerative diseases (77). The three major clinical types are classified by the involvement of primary central nervous system (CNS) disease. Type 1 GD, the most common variant, has a non-neuronopathic presentation characterized by the presence of hepatosplenomegaly, orthopedic complications, and blood disorders. Types 2 and 3 are characterized by the presence of primary neurologic disease distinguished by their rapid vs. gradual progressive course, respectively (78). PD is present in 4% of type 1 GD. Even in cases which the PD diagnostic criteria are not met, over 20% of GD patients were found to display at least one parkinsonian finding (79).

Evidence linking GBA mutations to the clinical characteristics of PD, including disease phenotype, progression, and prognosis, has been extensively documented. At the point of diagnosis, the clinical presentation of GD patients with PD can be almost identical to that of idiopathic PD (6). The most commonly presenting feature is an asymmetric resting tremor, although postural instability and gait difficulties are also relatively frequent (7). However, GBA carrier status has a significant impact on the natural history of PD. Patients reported an earlier age of symptom onset and severe motor impairment (8–10). Genetic polymorphism has been shown to influence motor progression (6). There is a higher prevalence of dementia and a distinct pattern of cognitive deficits, characterized by greater impairment in memory, executive function and visuospatial abilities (8, 9, 11, 12). The accelerated cognitive decline is indicated to relate the GBA mutation severity (6, 9, 11–14). Other non-motor clinical features are also highly prevalent, with the most common being anosmia and dysautonomia, as well as REM sleep disorder, depression, anxiety, and psychotic features presenting as hallucinations (8, 9, 15). A 2-fold increase in mortality risk in GBA-carriers has also been found (9).

Clinical reports of an association between GD and parkinsonism instigated further investigation into the mechanistic and genetic overlap between the two syndromes. The fundamental finding that a GBA1 gene mutation is a significant risk factor for PD development is of significant interest (10, 80, 81). Mutations in GBA1 amongst PD patients are common and reported as the most important risk factor yet discovered for PD, present in 3.5–6.7% of the PD population (6, 10, 82, 83). It is reported as the first finding of a genetic locus shown to influence motor progression in PD, possibly leading to its role as a prognostic marker in the future (6). Most analyses have focused on the most common pathogenic mutations in GBA, N370S and L444P variants, which represents up to 50% of the genetic alterations (10, 13, 80, 84, 85). In fact, it is reported that over 90% of GD-PD patients carry at least one N370S mutation (86). L444P mutation has recently been found to have a highly detrimental effect, responsible suspending the production and destabilization of GCase (35). Carriers with severe GBA mutation have been found to have a higher risk of PD development, with earlier onset of symptoms (87). For example, patients with the GBA mutation 84insG, a severe mutation leading to decreased GCase enzyme activity, are at higher risk of developing PD compared to milder mutations such as N370S (36).

The overexpression of the poorly functional GBA1, leading to reduced GCase levels and subsequent accumulation of its lipid substrate GlcCer, is found to be linked with increased a-syn accumulation and subsequent neuronal cell death. The accumulation of GlcCer contributes to the destabilization of a-syn tetramers and increase the susceptibility of human dopaminergic neurons to the cytotoxic exposure to pathologic a-syn fibrils (37–41). It was also demonstrated that GBA1 dysfunction and GCase depletion enhances the propagation of a-syn aggregations among neural cells, which is associated with the progression of PD (42). The pathologic implication of reduced GCase levels was supported by the finding that the direct administration of GCase to the brain resulted in a reduction of a-syn levels and an improvement of parkinsonian symptoms (38, 88–90).

Conversely, reduced levels of GCase were also associated with increased a-syn in idiopathic PD patients without GBA mutations, indicating that a-syn can independently inhibit GCase enzyme activity (36, 91–93). A-syn's contribution to decreased GCase activity is through its disruption of GCase and other lysosomal hydrolases trafficking to lysosomes, further contributing to lysosomal dysfunction and neuronal dyshomeostasis (38). These findings support the “gain of function” hypothesis whereby mutated GCase promotes a-syn aggregation, accelerating lewy body formation and neuronal loss (94). In summary, the possible mechanism indicated to underlie a-syn accumulation and PD development is the perpetual cycle in which GCase depletion increases the level of a-syn accumulation, which in turn inhibits the regular function of GCase and causes further aggregation of a-syn (40).

An alternate theory is the association between heterozygote GBA mutations and dysregulations in the protein degradation systems, which have been implicated as a common mechanism underlying neurodegenerative diseases. PARK2 gene mutations encoding parkin, the E3 ubiquitin ligase, has been identified as a significant cause for an autosomal recessive form of PD (43). Parkin is involved in the mediation of the proteasomal degradation of misfolded mutant GCase. Parkin was suggested to be occupied by ubiquitination of misfolded GCase in GD carriers, causing accumulation of other natural Parkin substrates, which is detrimental to dopaminergic neuronal survival, contributing to their demise and subsequent PD development (44). Dopaminergic cells expressing mutant GCase are particularly susceptible to apoptotic stimuli (95). Further investigation is necessary to unravel the mechanism of the bidirectional relationship between GBA1 mutation and a-syn and the effect of GBA mutations in misfolded protein accumulation leading to cell damage.

#### Niemann-pick disease

Niemann-Pick Disease (NPD), a neurovisceral LSD, presents with a broad clinical spectrum ranging from a rapidly fatal disorder in neonates to a chronic neurodegenerative disease later in life. The two distinct metabolic abnormalities include, firstly, a deficiency in the enzyme sphingomyelinase (ASM) resulting from mutations in the SMPD1 gene accounting for types A and B NPD. Growing evidence suggests they represent opposite ends of the clinical spectrum with regard to the disease severity and neurologic involvement. The second category accounting for NPD type C (NP-C) involves a dysfunction in cholesterol transport due to NPC1 and NPC2 gene mutations (96, 97). NP-C is of particular interest in this discussion due to parkinsonism symptoms reported among heterozygeous carriers of NPC1 gene mutations and the association between impaired cholesterol metabolism in the brain and neurodegeneration (98, 16).

Presentations of parkinsonian symptoms have been reported in several cases of patients with mutated NPC1, encoding a lysosomal cholesterol transporter that regulates vesicular trafficking. Parkinsonian symptoms are less frequent manifestations compared to the more prevalent deep brain signs such as cerebellar ataxia and supranuclear opthalmoplegia in NP-C (99). Presenting motor features include dysarthria, tremor, rigidity, bradykinesia, postural instability, as well as stooped posture and festinating gait, impaired fine motor skills, and hypomimia. Autonomic dysfunction, including orthostatic hypotension, constipation, urinary urgency, and impotence, was also present along and sensory symptoms of hyposmia and difficulty in concentration (16, 17).

Alterations in the autophagy-lysosome pathway were suggested as a contributory pathophysiological factor for PD in NP-C. Dysregulations in cholesterol trafficking resulting in increased cholesterol deposition in neuronal cells have been observed in both NP-C and in PD (45). Evidence supporting the positive correlation between high plasma cholesterol level and increased risk of PD development includes: (1) higher cholesterol derivatives in brains and plasma of PD individuals (100, 101), (2) elevated levels of oxidized cholesterol metabolites increasing levels of a-syn and accelerates its aggregation (102–104) (3) cholesterol inhibitors, such as statins, reduce the levels of a-syn in neuronal cells whereas exogenous cholesterol supplementation to neurons increase a-syn aggregation and reduces neuron growth (105). (4) *In vivo* evidence of increased vulnerability of midbrain dopaminergic neurons and exacerbation of PD symptoms to hypercholesterolemia (106).

These findings suggest that the defect in the metabolic pathways in NP-C could reveal pathogenic overlaps associated with aberrant a-syn aggregation and neurodegeneration in PD (45). However, controversial findings report a correlation between plasma cholesterol level, statin therapy, and the risk of PD development (107,–110). Reports supporting the notion that NPC1 variants are risk factors for PD also remain unclear, necessitating further clarification through extensive population genetic studies (16, 111).

#### Parkinsonism in lysosomal storage diseases

The significance of GBA1 mutations in PD development has led to further insights into the pathomechanism centered around the dysregulation of the autophagic-lysosomal pathway. The effects of PD mutations and a-syn aggregations which converge on the lysosomal degradation system indicates the significance of protein dyshomeostasis in the mediation of neurodegeneration in PD. Apart from the important connection with GD which have been extensively explored, associations between PD and other LSDs including GM1—(18–20) and GM2-gangliosidosis (112–114), neuronal ceroid lipofuscinosis (21, 22), and metachromatic leukodystrophy (115, 116) have been described, although these are not as well-documented due to the rarity of these conditions.

Remarkably, recent emerging data have suggested that the lysosomal connection in PD likely extends far beyond GBA1. A recent genome-wide association study (GWAS) found that several of the identified PD risk genes play an integral role in lysosomal biology and autophagy (117). Conversely, over half of PD cases in a cohort study were found to present with at least one damaging variant in the LSD gene. These results are consistent with the previous genetic analyses, revealing an important connection between genetic factors responsible for LSD and PD risk (118, 119). These findings indicate the possibility that LSD gene polymorphism may lead to lysosomal dysfunction, resulting in the accumulation of toxic substrates and increasing PD susceptibility (118).

### Disorders of metal metabolism

#### Wilson's disease

Wilson's Disease (WD), an inborn error of copper (Cu) metabolism, is caused by a mutation in the copper-transporting gene ATP7B. This autosomal recessive disease is characterized by excessive copper deposition, primarily within hepatocytes and in the CNS (120). Movement disorders, including tremor, choreiform movements, parkinsonism, and rigid dystonia are the characteristic neurological presentation of WD (121). The “wing-beating” tremor, a swinging movement of the upper extremity, in combination with dysarthria, is a classic observation, strongly suggesting a WD diagnosis (23, 24).

Parkinsonian symptoms of WD includes rigidity, bradykinesia, shuffling gait, hypophonic speech, hypomimia, and micrographia. The tremor observed may be resting, intentional, postural, or action-induced, though the most common form of tremor in WD is an irregular, jerky, dystonic tremor (23–25). Dysarthria is commonly observed as a result of parkinsonism or other neurological disorder including cerebellar and pyramidal dysfunctions in WD (26).

The indicated underlying pathomechanism of parkinsonian manifestations amongst heterozygotes for WD describes the process of prolonged free Cu and iron accumulation leading to oxidative damage in the basal ganglia. This stems from the frequent observation of Parkinson symptoms among WD patients as well as the reduced level of ceruloplasmin and Cu dyshomeostasis reported in PD patients (46). Cu dyshomeostasis in PD patients has been linked to mutations in the gene ATP7B, indicating a genetic overlap between the two disorders (47). Recent studies have found the interaction between Cu and a-syn to be involved in multiple pathways leading to neuronal damage, including increased oxidative stress, the production of toxic oligomers which eventually result in membrane breakage and enhanced a-syn fibrillation (47–49).

Ceruloplasmin deficiency leads to the binding between a-syn and excess free Cu, which acts as a ferrireductase and increases the availability of iron. The increase in iron deposition, occurring as a result may be involved in the underlying pathophysiology of PD onset and development (50, 51). The accumulation of both excess copper and iron may contribute to free radical generation and oxidative damage to the SNpc and the oxidative labile dopaminergic neurons (52). Evidence of nigro-striatal dopaminergic deficit can be found in WD subjects with neurological symptoms (120).

Further research is necessary to explore the role of copper, ceruloplasmin, and iron dyshomeostasis in PD and the possibility of an underlying heterozygote WD in some PD cases, in which help in an early diagnosis and in establishing promising therapeutic strategies.

#### Hemochromatosis

Hemochromatosis is an autosomal recessive disorder which leads to abnormal iron deposition in multiple organs, including the brain, leading to the development of neurodegenerative diseases. Rare reports of parkinsonian syndrome in hereditary hemochromatosis describes characteristic symptoms of resting tremor, bradykinesia, rigidity, and an asymmetric onset. Mixed resting and action tremor were also reported in one patient case (27–29).

Iron is essential in the maintenance of normal neurological function through its role in oxidative metabolism and the synthesis of neurotransmitters and myelin (122). As previously discussed, the dysregulation of iron metabolism has been suggested to contribute to the risk of PD development. This is further evidenced through the observation of altered iron levels in the SNpc of PD patients through MRI findings and postmortem samples (27, 123–125). Increased intracellular iron promotes the degeneration of dopaminergic neurons via several suggested pathways, in addition to the enhancement of oxidative stress as previously described (53). Iron dysregulation has been suggested to play a role in the dysfunction of the ubiquitin-proteasome system. The generation of reactive oxygen species (ROS) and enhancement of protein aggregations exaggerates proteasome dysfunction, resulting in the impairment of the ubiquitin-proteasome system, leading to neuronal injury (54). Iron has also been studied to promote a-syn aggregation and cell-to-cell transmission by inhibiting transcription factor EB (TFEB)-mediated autophagosome-lysoosme fusion (55). Iron induces alterations in Parkin solubility, resulting in its intracellular aggregation. The consequential depletion in functional Parkin leads to reduced proteasomal activities and ultimately, increased cell death (56).

Furthermore, recent data has demonstrated the neuroprotective role of iron chelators against dopamine neuronal degeneration and suggests its therapeutic potential for PD treatment (126, 127). Although the theoretical risk of increased parkinsonism exists with disorders of iron regulation, the association between hemochromatosis and its genetic mutations, levels of iron, and PD has conflicting results, necessitating further extensive population and genetic studies (27, 128, 129).

### Disorders of amino acid metabolism

#### Phenylketouria

Phenylketouria (PKU), a prevalent inherited defect in amino acid metabolism, is characterized by an underlying deficiency in phenylalanine hydroxylase, the hepatic enzyme that catalyzes the hydroxylation of phenylalanine (Phe) to tyrosine (Tyr) (130). The resulting accumulation of Phe leads to a neurological sequela of mental retardation, epilepsy, spastic paraparesis, and late-onset neuropsychological impairment in untreated PKU patients (131). Delayed neurological syndromes, including tremor and dystonia, are indicated to result from the hypomyelination in the CNS (30). Reports of parkinsonism, although exceedingly rare, have been documented as delayed manifestations amongst PKU patients (31). Presentations include the classical rigidity and bradykinesia, in addition to atypical tremor symptoms which presented both at rest and, at times, more prominently on action (30, 31).

It has been suggested that signs of parkinsonism may be secondary to the depletion of dopamine activity in the brain, a result of the reduction of brain Tyr, a dopamine precursor, as a biochemical consequence of PKU (31, 57, 58). This is supported by a report of decreased symptoms of parkinsonism after levodopa administration in a patient suffering from PKU (31). Further evidence has shown decreased dopamine levels in the frontal cortex of PKU subjects where cerebral dopamine deficiency has been implicated in the underlying pathophysiology of executive functioning deficits (132). On a different note, the exposure to high serum Phe during early life may result in adverse effects on early brain growth, including the development of brain dopamine. Chronic exposure to high Phe concentrations has been associated with oxidative stress, which may promote tissue damage and the elicit the onset and progression of neurological impairment in PKU (59, 60).

#### Maple syrup urine disease

Maple Syrup Urine Disease (MSUD), a disorder of branched-chain amino acid metabolism, manifests as toxic encephalopathy in early life. Treated adults commonly present with movement disorders, predominantly paroxysmal dystonia or ataxia. Patients with parkinsonism in MSUD present with bradykinesia on finger tapping with no resting tremor. These neurological symptoms were speculated to be secondary to the early cerebral damage and chronic reduction in levels of neurotransmitters (32).

Extensive neuronal loss in the pontine nuclei and SNpc in addition to various neurochemical changes were reported to contribute to the development of movement disorders (especially dystonia and ataxia) in MSUD (32). Free radical generation is found to be elicited in MSUD and is possibly involved in the pathophysiology of neurological dysfunction (61). Previous *in vitro* findings have indicated that accumulating metabolites caused the suppression of brain energy metabolism through the blockade of the respiratory chain, contributing to neurological impairment in patients (62). The depletion of dopamine levels and disruption of normal brain development which accompanied the presentation of limb dystonia and gait abnormalities in MSUD mice models has also been reported (63).

#### Methylmalonic acidemia

Methylmalonic acidemia is an autosomal recessive disorder of branched chain amino acid metabolism, caused by deficient activity of methylmalonyl-coenzyme A mutase. While primarily recognized as a pediatric condition, significant multi-systemic morbidity may be present in patients who reach adulthood (133). Among other neurological manifestations, movement disorders including dystonia, chorea, myoclonus, and tremor have been reported (134, 135). Although parkinsonian symptoms are less well-documented in this disorder, it is important to discuss the role of methylmalonic acid (MMA) in neurological dysfunction. Han et al. reported the toxic effects of MMA on neurons and its contribution to neuronal apoptosis (64). Mitochondrial oxidative damage and increase oxidative stress has also been reported in the involvement of neuronal apoptosis caused by MMA in the pathoetiology of PD (64, 65). Furthermore, a positive correlation has been found between serum MMA and the severity of neuropathic pain in amongst PD patients, suggesting its use as a potential marker in assessing PD peripheral neuropathy (33).

### Inherited mitochondrial disorders

Mitochondrial diseases are a heterogeneous group of disorders with multi-systemic involvement, from musculoskeletal to cardiac to gastrointestinal presentations. The spectrum of mitochondrial pathologies has been reported to result from mutations in the mitochondrial DNA which led to impaired oxidative phosphorylation and dysfunction of the mitochondrial respiratory chain (136). Growing evidence supports the role of mitochondrial dysfunction in the pathogenesis of both the sporadic and familial forms of the PD. Mitochondrial disorders should be particularly suspected in PD patients with extra-cerebral manifestations. Signs and symptoms of parkinsonism reported among these patients include masked faces, mental impairment, rigidospasticity, slowed movements, and gait disturbances (34). Finsterer et al. reported the observation of parkinsonism in 12% of patients with mitochondrial disorders (137). Another study found parkinsonism to be the most common movement disorder among patients with mitochondrial disease, presenting in 43% of research subjects (138). Conversely, 6% of patients with PD have been reported to present with phenotypic features of mitochondrial disorders during the course of their disease (34, 137).

The role of mitochondrial dysfunction in the pathomechanism of PD was initially observed through the findings of induced parkinsonism following the administration of the neurotoxin 1-methyl-4-phenyl-1,2,3,4-tetrahydropyridine (MPTP), an inhibitor of the mitochondrial respiratory chain (MRC) complex I (139, 140). This led to findings of complex I deficiency in the PD brain within the substantia nigra and other regions such as the frontal cortex (66, 141). The inhibition of MRC complex I resulted in enhanced production of ROS and increased oxidative stress, ultimately leading to cellular damage of the dopaminergic neurons in the SNpc (142).

Genetic evidence has further supported the common pathway between PD and the maintenance of mitochondrial function in dopaminergic neurons (143). A GWAS has identified genes sets which regulate pyruvate metabolism and the electron transport chain (ETC) which are under-expressed in the dopaminergic neurons of the SNpc of PD patients (67). Mutations or polymorphisms in several genes (including a-syn, parkin, PINK1, LRRK2, DJ-1) have implicated mitochondrial dysfunction as a prominent cause for PD pathogenesis (65, 68–74). For example, a recent study has shown that mutations in the PINK1 gene, resulting in a shortage of energy production, was implicated as an underlying cause of dopaminergic neuronal damage and subsequent movement difficulties associated with PD (75).

In addition to the oxidative stress and genetic polymorphism which has long been a focus in PD research, recent studies suggest that the disruption in the breakdown of MIRO (a protein connecting the outer mitochondrial membrane to microtubules) may contribute to the impaired process in the cleranace of damaged mitochondria. This reduced degradation of MIRO and the delay in the dysfunctional mitochondrial clearance appears to be a common characteristic of familial and sporadic PD (76).

## Conclusion and future directions

The clinical convergence between PD and IMDs has instigated further research into the overlapping genetic and biochemical dysregulations between the two conditions. The discovery of shared pathways of an unbalanced homeostasis continues to expand our understanding of the clinical implications of oxidative stress and aberrant protein accumulation on the process of neurodegeneration. Despite the substantial progress that has been made, much work remains to determine the validity and strength of the proposed link between IMDs and PD. Further genetic and biochemical studies may provide insights into risk factors that synergistically favor the development of parkinsonism in particular subsets of IMDs patients. The better understanding of how these factors interact and contribute to the development of parkinsonism may point to clues surrounding pathogenesis-targeted treatment. This includes the possibility of a prognostic marker to establish prompt diagnoses, leading to the availability of genetic counseling and promising therapeutic strategies which may alleviate the onset and progression of this disorder.

## Author contributions

NL Project administration, Supervision, Visualization, Writing review, and editing. PI Supervision, Writing review, and editing. MH and KP writing review and editing.

### Conflict of interest statement

The authors declare that the research was conducted in the absence of any commercial or financial relationships that could be construed as a potential conflict of interest.
